# Association between KSHV-Specific Humoral and T Cell Responses with Recurrence of HIV-Associated Kaposi Sarcoma

**DOI:** 10.3390/tropicalmed9060134

**Published:** 2024-06-18

**Authors:** Marie-Claire Mukasine, Gina Mulundu, Musonda Kawimbe, Keagan Mutale, Chibamba Mumba, Salum J. Lidenge, Owen Ngalamika

**Affiliations:** 1 Department of Pathology and Microbiology, University of Zambia School of Medicine, Lusaka P.O. Box 50110, Zambia; mukasinemarieclairef@gmail.com (M.-C.M.); ginamulundu0904@gmail.com (G.M.); chibambamumba@gmail.com (C.M.); 2 HHV8 Research Molecular Virology Laboratory, University Teaching Hospital, Lusaka P.O. Box 50110, Zambia; kawimbemusonda@gmail.com (M.K.); keaganmutale1234@gmail.com (K.M.); 3 Ocean Road Cancer Institute, Dar es Salaam P.O. Box 3592, Tanzania; sjlidenge@yahoo.co.uk; 4 Department of Clinical Oncology, Muhimbili University of Health and Allied Sciences, Dar es Salaam P.O. Box 65001, Tanzania; 5 Dermatology and Venereology Division, University Teaching Hospital, University of Zambia School of Medicine, Lusaka P.O. Box 50110, Zambia

**Keywords:** Kaposi sarcoma, Kaposi sarcoma-associated herpes virus, antibodies, T cell responses, recurrence, sustained remission

## Abstract

Kaposi sarcoma (KS) is an AIDS-defining angio-proliferative malignancy, with the Kaposi sarcoma-associated herpes virus (KSHV) as its etiologic agent. Upon treatment with chemotherapy, a proportion of HIV-associated KS patients experience disease recurrence within a few months of completing treatment. We aimed at determining whether KSHV-specific adaptive immune responses were associated with KS recurrence upon complete remission. We conducted a prospective cohort study. The primary outcome was the recurrence of HIV-associated KS. An immunofluorescence assay was used to determine anti-KSHV antibodies, an enzyme-linked immunospot was conducted for T cell responses, PCR was carried out to determine KSHV status, and flow cytometry was used for CD4 counting and immunophenotyping. KSHV detection in PBMCs was high and not associated with KS recurrence-free survival (*p* = 0.29). Anti-KSHV antibody titers were high and not associated with recurrence-free survival (*p* = 0.63). KSHV-specific T cell responses dropped from baseline levels among individuals with recurrence, but the drop was not statistically significant. Individuals experiencing KS recurrence had a significantly higher proportion of T cell subsets expressing PD1, while those with sustained remission had a significant increase in CD4 T cell counts from baseline levels during the follow-up period (*p* = 0.02). Anti-KSHV antibodies are not a good correlate of protection from KS recurrence. T cells in individuals experiencing KS recurrence hadhigh PD1 expression, while an increase in CD4 counts was associated with sustained KS remission.

## 1. Introduction

HIV-associated Kaposi sarcoma (Epidemic KS) is an AIDS-defining angio-proliferative malignancy with high recurrence rates upon treatment with chemotherapy [1,2]. Factors associated with the high recurrence rates of epidemic KS upon treatment with chemotherapy are not fully established. In a previous study, we observed that cytokines and chemokines including IL6 and CXCL10 correlate with KS recurrence [2]. We also observed in the same study that the continued detection of HIV viral loads in plasma, despite treatment with antiretroviral therapy and HIV viral load suppression, was associated with an increased risk of KS recurrence. Other studies have also observed that cytokines and chemokines including IL6, IL10, and CXCL10 are higher in epidemic KS patients compared to the controls [3].

Kaposi sarcoma-associated herpes virus (KSHV) is the etiological agent for all types of KS. KSHV is highly prevalent in some parts of the world such as Sub-Saharan Africa (SSA), where seroprevalence rates are above 50% in the adult populations of most countries [4], and has a low seroprevalence in most developed countries with reported seroprevalence rates below 10% [5]. This partly explains the high incidence and prevalence of KS in many SSA countries compared to developed countries [6]. KSHV is necessary but not sufficient for KS development, progression, and recurrence. Other factors including HIV infection, immunosuppressive therapy, and male gender are known to increase the risk of KS development and/or progression [7,8].

Anti-KSHV antibodies have been observed to increase in titer when a KSHV-infected individual develops KS [9]. In previous studies, it has been observed that KS patients have significantly higher anti-KSHV antibody titers than KSHV-seropositive HIV^+^ and HIV^−^ without KS [10]. Other studies have also observed and reported an increased risk of KS development with increasing antibody titers against the KSHV latency-associated nuclear antigen (LANA) [11,12]. Therefore, these anti-KSHV antibodies may not be a good correlate of protection from the development and progression of KS. However, whether serum anti-KSHV antibodies provide secondary protection from KS re-development upon successful treatment with cancer chemotherapy remains to be determined.

The increased risk of KS development upon a decline in T cell immunity is a clear indication of the importance of T cell immunity in the control of KSHV and/or KS [13]. Previous studies have reported that T cell responses against KSHV are weak and lack immune dominance [14]. A study by Robey et al. shows that genes expressed in the early and late phase of the lytic cycle of KSHV (including ORF33, K1, and K8.1) are frequently recognized T cell targets [15]. T cell responses have also been associated with response to treatment. Patients with HIV-associated KS experiencing a regression of KS lesions while on antiretroviral therapy (ART) have been observed to have better KSHV-specific T cell responses and undetectable KSHV in plasma compared to progressors [16]. However, it remains to be established whether KSHV-specific T cell responses in individuals in remission for HIV-associated KS are correlates of protection from KS recurrence.

In this study, we sought to longitudinally investigate whether the presence and/or titer of anti-KSHV antibodies and anti-KSHV-specific T cell responses correlate with the recurrence of HIV-associated KS after successful treatment with chemotherapy.

## 2. Materials and Methods

### 2.1. Study Design and Participants

We conducted a prospective cohort study, from January 2019 to August 2023, on 47 consenting participants at the University Teaching Hospital (UTH) in Lusaka, Zambia. The study participants were individuals who were in clinically determined to be in complete remission for HIV-associated KS after treatment with chemotherapy. They were recruited by convenience sampling at least one month after the last dose of chemotherapy and not more than 2 months after and had no signs of KS at the time of recruitment into the study. All the participants were on ART for at least 6 months at the time of recruitment and were previously diagnosed with mucocutaneous but not visceral KS. The participants received doxorubicin, vincristine, and bleomycin (ABV) as their chemotherapy regimen at the dermatology department at the UTH. At baseline, sociodemographic information (including age, gender, smoking status, and alcohol) was collected, and a thorough clinical assessment by clinicians trained in the diagnosis and management of KS at the dermatology department at the UTH was conducted. We collected venous blood for the laboratory assays at baseline and at all follow-up visits. A portion of the fresh whole blood was subjected to flow cytometry for CD4 counting, immunophenotyping, and determining the expression of immune checkpoints. The rest of the blood was centrifuged to obtain serum for determining HIV viral loads and anti-KSHV antibody detection and quantification. Density gradient centrifugation was then conducted to isolate PBMCs for KSHV detection and IFNγ enzyme-linked immunospots (ELISpots). Participants were followed up at least once every 2–3 months for a period of one year or until they developed disease recurrence. Recurrence was determined as the clinical re-appearance of one or more KS lesions in a patient that had a regression of all KS lesions after treatment with chemotherapy. At least 2 clinicians at the dermatology clinic confirmed the re-development of KS lesions.

### 2.2. HIV Viral Loads and CD4 Counts

All participants were HIV-positive. We measured HIV-1 plasma viral load on the Hologic Panther (Hologic, Marlborough, MA, USA) using the Aptima HIV-1 Quant Dx Assay kit (Hologic) according to the manufacturer’s protocol. We also determined CD4 counts in all the study participants using the BD TriTest kit (BD Biosciences, San Jose, CA, USA) on a BD FACSCalibur instrument (BD Biosciences). These parameters were assessed at baseline and at the final follow-up visit.

### 2.3. KSHV Detection in PBMC

We extracted DNA from peripheral blood mononuclear cells (PBMCs) using a commercial DNA extraction kit (QIAamp DNA Mini Kit, Qiagen, Germantown, MD, USA) according to the manufacturer’s protocol. We then prepared a PCR reaction and performed a PCR reaction on a thermocycler using Taq polymerase enzyme and KSHV ORF-26 forward (5′-AGCCGAAAGATTCCACCAT-3′) and reverse (5′-TCCGTGTTGTCTACGTCCAG-3′) primers. This was followed by gel electrophoresis to determine the presence of amplified KSHV amplicons. PCR for the housekeeping gene beta actin was carried out as a control for the presence of cellular DNA using forward (5′-TTCTACAATGAGCTGCGTGT-3′) and reverse (5′-GCCAGACAGCACTGTGTTGG-3′) primers. In addition, a known KSHV-positive sample and a negative control were included as experiment controls. Negative samples after the first reaction were subjected to a second PCR reaction using nested forward (5′-CGAATCCAACGGATTTGACCTC-3′) and reverse (5′-CCCATAAATGACACATTGGTGGTA-3′) primers. This was followed by another gel electrophoresis to determine the presence of KSHV in the samples. Samples that were positive after the first and, if necessary, the second PCR reaction, were considered KSHV-positive.

### 2.4. Anti-KSHV Antibody Detection and Titers

We performed an immunofluorescence assay to detect and measure the levels of anti-KSHV antibodies in participants’ serum, as previously described [17]. This immunoassay detects antibodies against multiple lytic and latent KSHV open reading frames following the reactivation of the virus using tetradecanoyl phorbol acetate at a final concentration of 20 ng/mL for 72 h and permeabilization of the membranes using 0.1% Triton X-100. A 1:40 dilution of clear patient serum and positive control serum was conducted using 0.1% Tween-20 in 1X PBS blocking solution. We then added 15 µL of diluted serum to pre-labeled BC3 slides and incubated the slides at 37 °C for 30 min. After incubation, slides were then washed with 1X PBS and air-dried. Moreover, a 5-fold diluted mouse monoclonal anti-human IgG antibody (ATCC, Manassas, VA, USA) was then added to the wells, followed by incubation at 37 °C for 30 min and washing. Finally, donkey anti-mouse IgG (Jackson ImmunoResearch Laboratories, West Grove, PA, USA) was diluted 100-fold, added to the wells, incubated at 37 °C for 30 min in a humidity chamber, and washed. Slides were then stained with 0.004% Evans Blue solution, rinsed, air-dried, and mounted with Fluoromount aqueous mounting media. The slides were then examined under a fluorescence microscope using dual FITC and Tritc filters. The stained IFA slides were examined by three independent readers with a Nikon Eclipse 50i fluorescence microscope and positive cells appeared green in color. A well was only considered positive or negative if at least two readers reached the same result independently. Samples that were positive for an anti-KSHV antibody at 1:40 dilution were further serially 2-fold diluted and re-assayed to determine end-point positive dilution regarded here as the anti-KSHV antibody titer. Anti-KSHV antibody detection and titers were measured at baseline and at the final follow-up visit.

### 2.5. Anti-KSHV T Cell Responses

**Figure A1:**
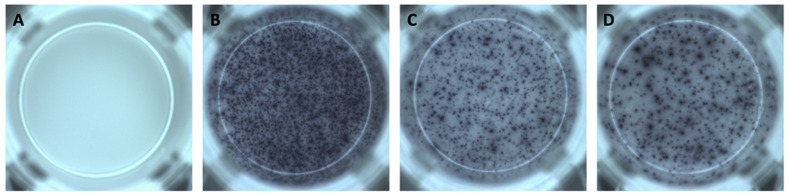
*ELISpot results*. The figure shows the appearance of the spots after the assay. DMSO was used in place of peptides as a negative control (**A**), CD3 monoclonal antibody was used as a positive control and had the most number of spots (**B**), a pool of CMV/EBV peptides were used as an additional control with a number of spots in the wells (**C**), while K8.1 peptides were used for detecting responses against KSHV (**D**).

We conducted IFNγ ELISpots to detect KSHV-specific T cell responses in 35 study participants (Figure A1). ELISpots were conducted on a subset of the cohort as the assay was developed after the study had already commenced, and we only performed the assay on freshly isolated PBMCs and not cryopreserved cells. We conducted a 2-day experiment in duplicate for each participant for each study visit. On the first day, we added 250,000 live whole PBMCs to 96-well precoated plates from MABTECH AB (Nacka Strand, Sweden). We then added 2 µg/mL of 15-mer KSHV K8.1 (Peptides & Elephants, Hennigsdorf, Germany) peptides with 11 amino acid overlaps with the wells. Cell counting was conducted using an automated counter (Countess II FL, Invitrogen, Carlsbad, CA, USA). Anti-CD3 was used as a positive, DMSO was used instead of peptides in the negative control wells, and 0.1 µg/mL of an anti-CD28 monoclonal antibody was used for co-stimulating T cells in all the wells, including positive and negative controls. We also incorporated a pool of CMV and EBV peptides as an additional control for each participant. After mixing the cells and peptides, we put the 96-well plate placed in a 37 °C humidified incubator with 5% CO_2_ for about 24 h. After the incubation, we developed the spots according to the manufacturer’s protocol.

The final part of spot development was carried out over a 10 to 12 min period in all the participants. After 12 min, we inhibited further spot development with running water. We then left the plate to dry in the dark overnight and counted the spots the following day on an AID ELISpot reader (Autoimmun Diagnostika, Straßberg, Germany). We standardized spot characteristics for all the participants at a minimum intensity of 3, minimum size of 20, and minimum gradient of 1. Spot forming units above 55 and 4× higher than the background were regarded as a positive response.

### 2.6. Flow Cytometry

**Figure A2:**
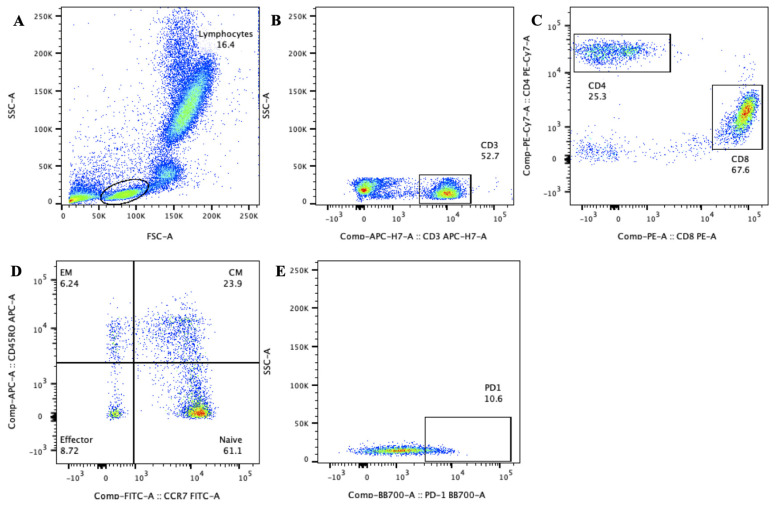
*Gating strategies on FlowJo analysis*. Forward and side scatter was used to visualize the different cell populations, then a gate was created on the lymphocyte population (**A**). We then used the lymphocyte population to gate on CD3^+^ (T cells) population (**B**), followed by selecting and gating on the CD4^+^ and CD8^+^ cells from the CD3+ population (**C**). CCR7 and CD45RO were then used to identify naïve central memory (CM), effector memory (EM), and effector cells among both CD4^+^ and CD8^+^ T cells (**D**,**E**). Finally, we gated on PD1^+^ cells among all the T cell subpopulations.

Flow cytometry was conducted concurrently with the T cell assay, on fresh whole blood, to immunophenotype the T cells and determine the expression of markers of exhaustion on the T cell subsets. We stained 100 µL of whole blood with CD3-APC-H7 (BD Biosciences, San Jose, CA, USA; RRID: AB_1645475), CD4-PE-CY7 (BD Biosciences; RRID:AB_1727475), CD8-PE (Miltenyi Biotec, Macquarie Park, NSW, Australia; RRID:AB_2726261), CCR7-FITC (Miltenyi Biotec; RRID:AB_2752108), CD45RO-APC (BD Biosciences; RRID:AB_398673), and PD1-BB700 (BD Biosciences; RRID:AB_2738827) for 20 min in the dark. After the staining, we lysed the red blood cells with FACS lysing solution (BD Biosciences). After washing the cells, we resuspended the stained cells in 350 µL PBS. Flow cytometry was performed on a 6-color BD FACSVerse instrument (BD Biosciences). Compensation was conducted, and fluorescence-minus-one controls (FMOs) were used to guide on gating boundaries (Figure A2). We set up the experiment to collect at least 10,000 events in the lymphocyte gate. FlowJo software (version 10) was used to analyze the data. The data were then exported to Excel as proportions of T cell subsets and cells expressing the immune checkpoint PD1, followed by statistical analysis using STATA.

### 2.7. Statistical Analysis

STATA version 17 was used for the statistical analyses (StataCorp, College Station, TX, USA). We used descriptive statistics to analyze the baseline characteristics. Continuous variables are expressed as the median and interquartile range, while categorical variables are expressed as percentages. The chi-squared test was used to determine association between the binary outcome and binary predictors. The Wilcoxon rank-sum test was used to compare continuous variables between the two outcome groups. Baseline compared to follow-up continuous variables within groups were compared using the Wilcoxon matched-pairs signed-rank test. The Kaplan–Meir survival estimate was used to estimate and visualize the recurrence-free survival. Univariate and multivariate cox regression was also used to investigate the effect of variables on time to KS recurrence. The inclusion of variables in the multivariate models was based on low *p* values (<0.2), or variables expected to have a confounding effect. However, due to the low number of participants, we could only build models with very few variables in order to avoid overfitting the model. We also used the random intercept and slope model to analyze T cell response results that were collected repeatedly over time. This was a multilevel model that accounted for repeated observations in each participant and allowed participants to have their own intercepts and slopes (change in T cell responses over time). *p* values < 0.05 were considered statistically significant. CD4 counts, HIV viral loads, KSHV detection in PBMCs, and anti-KSHV antibody detection and titers were carried out and analyzed at baseline and final follow-up time-points. Immunophenotyping and anti-KSHV T cell responses were carried out and analyzed repeatedly at multiple time-points.

## 3. Results

### 3.1. Baseline Characteristics

We recruited 47 participants in this study, with 44.7% having KS recurrence during the follow-up period. Baseline sociodemographic characteristics including age, gender, smoking status, and alcohol consumption were similar between individuals with sustained remission and those who had a recurrence. There were high PBMC KSHV detection rates and anti-KSHV antibody titers at baseline in both outcome groups. The rest of the baseline clinical, sociodemographic, and laboratory parameters are shown in Table 1. KSHV-specific T cell responses and the assessment of immune checkpoints were analyzed and conducted on 35 of the study participants because the assay became available after recruitment had already commenced. Characteristics of the subgroup are shown in Table A1. Among the with sustained remission, less than a quarter (6/26) were lost to follow-up.

### 3.2. Factors Associated with Recurrence-Free Survival

**Figure 1:**
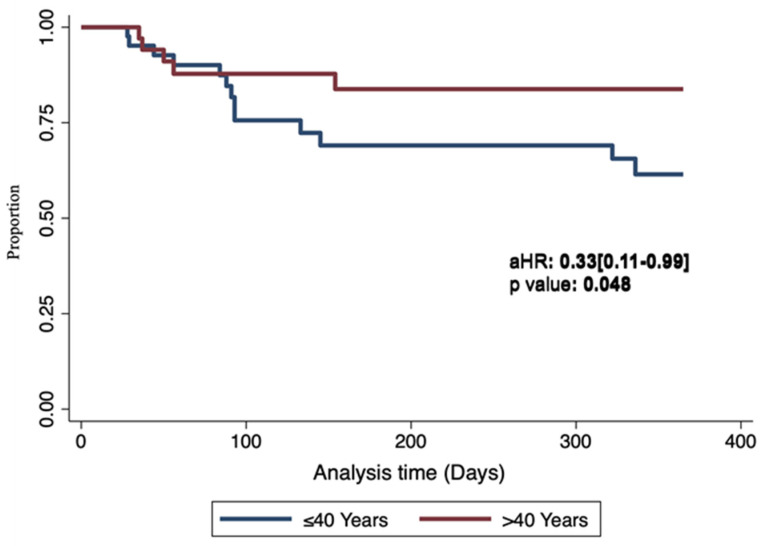
*Recurrence-free survival by age*. Age was categorized as >40 years (N = 18) and ≤40 years (N = 29). After adjusting for plasma HIV viral load detection, an age > 40 years was associated with a better KS recurrence-free survival.

As seen from the univariate analysis, none of the clinical, sociodemographic, HIV-related viral and immunological factors, and KSHV-related parameters (KSHV detection in PBMCs and anti-KSHV antibody titers) were associated with recurrence-free survival (Table 2). After adjusting for plasma HIV viral detection at baseline, older participants above 40 years of age had a significantly better recurrence-free survival than younger patients 40 years of age and below (Figure 1). This age was chosen for categorizing age because of the expected 90% involution of the thymus, important for T cell maturation, by the age of 40 years [18]. On the other hand, there was a borderline higher risk of KS recurrence in individuals that had detectable plasma HIV viral loads at baseline after adjusting for the categorized age (aHR: 2.53 [0.99–6.49]; *p* = 0.053). Subsequent analysis showed that older individuals had significantly higher CD4 counts than the younger ones (254 cells/µL vs. 207 cells/µL; *p* = 0.033). Upon adjusting for CD4 counts, we observed no association between categorized age and KS recurrence (aHR: 0.42 [0.13–1.39]; *p* = 0.155).

### 3.3. Association of KSHV Viral Detection and Anti-KSHV Antibodies with KS Recurrence

In order to determine whether KSHV detection in PBMCs is associated with HIV-associated KS recurrence, we investigated KSHV in participants’ PBMCs. At baseline, there was a higher detection of KSHV in PBMCs of individuals that ultimately had a recurrence compared to those with sustained remission. However, this difference was not statistically significant (85.7% vs. 70%; *p* = 0.29). There was a statistically insignificant lower detection rate of KSHV in PBMCs of individuals with a recurrence compared to those with sustained remission at the time of the primary response (64.3% vs. 76.9%; *p* = 0.47).

**Figure 2:**
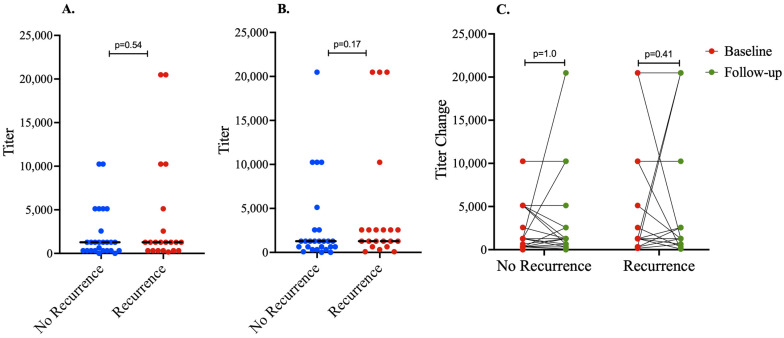
*Anti-KSHV antibody detection and titers.* The median anti-KSHV antibody titers were similar and high (1:1280) at both baseline (**A**) and follow-up (**B**). The titers were not significantly different between individuals with and without recurrence. Also, there was no significant change in anti-KSHV antibody titers during the follow-up period in both groups (**C**). The anti-KSHV antibody assays were performed at baseline and at the last follow-up visit.

We investigated whether or not anti-KSHV antibody detection and/or titers are a correlate of protection from HIV-associated KS recurrence upon successful treatment with chemotherapy. The median anti-KSHV antibody titers were high at baseline (1:1280) (Figure 2A) and remained high at the time of the determination of either recurrence or sustained remission (1:1280), with no statistically significant difference between the two groups (Figure 2B). In addition, there was no significant difference in anti-KSHV antibody titers between baseline and follow-up within the two groups (Figure 2C).

### 3.4. Association between CD4 Counts and HIV-Associated KS Recurrence

**Figure 3:**
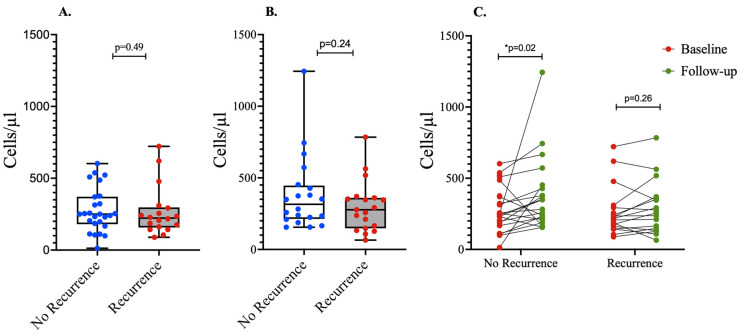
*Association between CD4 counts and KS recurrence*. CD4 counts were not significantly different between the two groups at baseline (**A**) and follow-up (**B**). However, there was a significant increase in CD4 counts in the individuals who had sustained remission during the follow-up period (**C**). CD4 counts were assessed at baseline and at the last follow-up visit. * = statistically significant *p* value.

Since CD4 counts are a measure of the detrimental effects of HIV infection on the immune system, we further investigated whether changes in the CD4 counts associated with the recurrence of HIV-associated KS. At baseline, there was no significant difference in the median CD4 counts between the two outcome groups (Figure 3A). At the time of the determination of the primary outcome, the median CD4 counts were higher in the group with sustained remission than the group with KS recurrence. However, this difference was not statistically significant (Figure 3B). On the other hand, we observed a significant increase in median CD4 counts from baseline counts at the time of the determination of the primary outcome in individuals with sustained remission (Figure 3C). The group experiencing KS recurrence had no difference in CD4 counts between the baseline and follow-up counts.

### 3.5. KSHV-Specific T Cell Responses, T Cell Exhaustion, and HIV-Associated KS Recurrence

**Figure 4:**
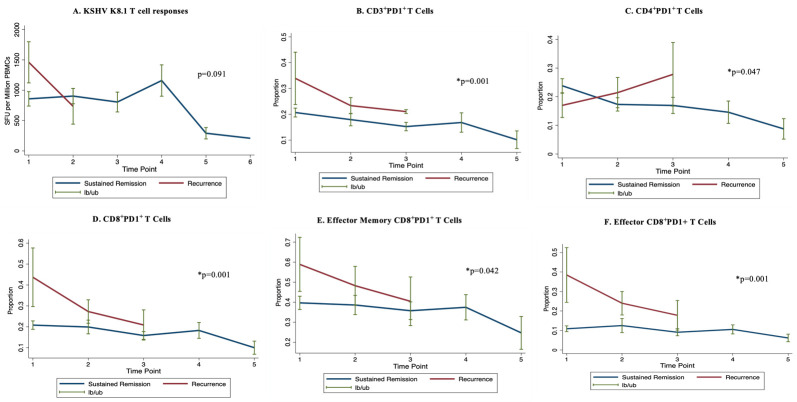
*KSHV-specific T cell responses and markers of exhaustion*. Statistically insignificant rapid decline in KSHV-specific T cell responses (against lytic antigen K8.1) in individuals with KS recurrence compared to those with sustained remission (**A**). Significantly higher proportions of CD4+ and CD8+ T cells expressing the immune checkpoint PD1 over time in individuals experiencing HIV-associated KS recurrence compared to the ones with sustained remission (**B**–**F**). lb = lower boundary; ub = upper boundary; PD1 = programmed cell death protein 1; time-point = follow-up visit; * = statistically significant *p* value.

The KSHV-specific T cell responses appeared to decline rapidly during the follow-up period leading to KS recurrence in individuals experiencing recurrence compared to individuals with sustained remission. However, this observation was not statistically significant (Figure 4A). On the other hand, we observed significantly higher proportions of CD4+ and CD8+ T cells and their subsets expressing the immune checkpoint PD1 in the peripheral blood of individuals experiencing KS recurrence compared to individuals with sustained remission over time (Figure 4B–F).

## 4. Discussion

The main objective of this study was to determine whether KSHV detection, anti-KSHV antibodies, HIV-related parameters (HIV viral load and CD4 counts), KSHV-specific T cell responses, and markers of T cell exhaustion are associated with the recurrence of HIV-associated KS upon achieving complete remission with chemotherapy. To the best of our knowledge, there were no studies on these factors and how they associate with KS remission at the time this manuscript was being written.

We observed that an age greater than 40 years was associated with a better recurrence-free survival. We did not observe any association between age and KS recurrence in a previous study [3], possibly due to the fewer number of study participants in the previous study. Also, age was analyzed as a continuous variable in the previous study, while it was categorized in the current one. The other possible explanation is our observation that individuals above 40 years of age had significantly higher CD4 counts than the ones who were 40 years of age and below. Upon further analyses, we observed no association between an age above 40 years and recurrence after adjusting for CD4 counts. The observation of lower CD4 counts in individuals above 40 years, and how this was associated with KS recurrence in our study, is supported by a previous study that showed that low CD4 counts during follow-up were marginally associated with a higher risk of KS recurrence [19].

Since KSHV is the etiological agent for KS, we sought to determine whether its detection in PBMCs is associated with KS recurrence. We observed a high (≥70%) detection rate of KSHV in PBMCs of individuals in both outcome groups at baseline. The group that had recurrence had a higher proportion of detection, but this was not statistically significant. In a previous study, the detection of KSHV DNA in plasma did not correlate with response to treatment [20]. Despite this study having a different design and detecting KSHV in plasma and not PBMCs, it still supports our observation that KSHV detection may not be a good marker of KS recurrence or sustained remission. Other studies in classical KS have reported a lower detection rate of KSHV (14.8%) in PBMCs after 6 months of achieving complete remission [21]. These differences may be due to higher replication and detection rates of KSHV in individuals with HIV compared to HIV-uninfected individuals [22,23]. Nevertheless, our sample size was limited in order to make definitive conclusions.

Anti-KSHV antibodies are known to be elevated in individuals with KS compared to KSHV-seropositive individuals without KS [9,24]. Our goal in this study was to investigate whether serum anti-KSHV antibodies persist, and if they do, whether their titers are protective against KS recurrence upon treatment with chemotherapy. We observed a high titer of anti-KSHV antibodies in both outcome groups. These anti-KSHV antibody titers remained high even at the time of KS recurrence and in individuals with sustained remission. Our findings are similar to a previous study that observed that anti-KSHV antibody responses before and after KS treatment were not associated with treatment response [20]. In a study by Tedeschi et al., it was observed that KSHV viral loads correlated with titers of anti-KSHV antibodies to lytic antigens [25]. Other studies have also reported the high seroprevalence (>90% detection rate) of anti-KSHV antibodies among patients with HIV-associated KS [26]. It has also been observed previously that ART initiation is associated with a rise in the plasma detection of anti-KSHV antibodies [27]. The continued antigenic stimulation by persistent KSHV detection and the fact that all our study participants were on ART for some time likely explains the high detection and titer of anti-KSHV antibodies in our cohort. Therefore, anti-KSHV antibodies may not be a good correlate of protection from the recurrence of HIV-associated KS after successful treatment with chemotherapy.

HIV infection and the immunosuppression associated with HIV infection are an important risk factor for KS development [28]. We found no association between HIV viral loads and HIV-associated KS recurrence. It is possible that because majority of the participants were on ART with suppressed HIV viral loads, we could not detect the association between viral load and KS recurrence. In addition, CD4 counts at baseline were low and not differential between the two outcome groups. However, individuals who had sustained remission had a significant increase in CD4 counts during the follow-up period. These findings support the importance of cell-mediated immunity for the control of KS and KSHV infection. Our observations are supported by studies that have reported a higher likelihood of poor outcomes among HIV-associated KS patients with low CD4 counts and higher risk of HIV-associated KS development among individuals with low CD4 counts [29,30]. Other observations in our study that may support the importance of T cell immunity in protecting against KS recurrence are the rapid decline of KSHV-specific T cell responses and the significantly higher proportions of T cells expressing the marker of exhaustion (PD1) in individuals experiencing recurrence compared to those with sustained KS remission. Some previous studies have observed and reported that the lytic reactivation of KSHV upregulates the expression of PD-L1, which is a ligand for PD1 [31]. While we observed higher PD1 expression on T cells, other studies have demonstrated higher PD1 expression on natural killer (NK) cells in KS patients [32]. More longitudinal studies on KSHV-specific T cell responses, with larger sample sizes, are required to definitively determine the role of KSHV-specific T cell responses in protecting against KS recurrence.

The inability to measure neutralizing antibodies was a major limitation of this study. However, previous studies have found no association between the presence and titer of KSHV-neutralizing antibodies and response to treatment [20]. Therefore, we do not expect that neutralizing antibodies are a correlate of protection from KS recurrence. Another limitation is that we did not measure anti-KSHV antibodies repeatedly over time, as they may have different patterns over time, and this could inform on the potential differences in trends between the two outcome groups over time. Other limitations in our study were that we did not quantify smoking and alcohol intake, including the initial extent of lymphedema and mucous membrane involvement prior to treatment with chemotherapy, which could limit our analyses of how these factors are associated with KS recurrence.

## 5. Conclusions

KSHV detection and anti-KSHV antibody titers are not associated with the recurrence of HIV-associated KS upon successful treatment with chemotherapy. A rise in CD4 counts is a good predictor of sustained remission for HIV-associated KS upon treatment with chemotherapy. A high proportion of CD4+ and CD8+ T cells expressing the immune checkpoint PD1 is associated with HIV-associated KS recurrence.

## Figures and Tables

**Table 1:** Baseline clinical and sociodemographic characteristics by KS recurrence status.

	Sustained Remission (N = 26)	KS Recurrence (N = 21)	*p* Value
Median Age (Years)	40 (30–48)	39 (31–41)	0.23
Males	61.5%	61.90%	0.98
Smoking	26.9%	33.30%	0.63
Alcohol	69.2%	57.10%	0.39
Lymphedema	34.6%	4/19 (21.1%)	0.32
Mucosa Involved	19.2%	2/19 (10.5%)	0.43
Duration on ART (Months)	24 (11–48)	18 (12–36)	0.65
KSHV Detected in PBMC at Baseline	70%	85.7%	0.29
Baseline Anti-KSHV Antibody Titer	1:1280 (320–2569)	1:1280 (320–2560)	0.54
HIV Viral Load Detected at Baseline	23.1%	42.9%	0.15
Baseline HIV Viral Load (Copies/mL)	0 (0-0)	0 (0–966)	0.12
Baseline CD4 Count (Cells/µL)	250 (185–370)	223 (162–293)	0.48
Median Follow-up (Days)	356.5 (336–390)	91 (50–154)	

**Table 2:** Factors at baseline associated with disease-free survival on univariate cox regression analysis.

	Crude Hazard Ratio [95% Confidence Interval]	*p* Value
Age	0.96 (0.91–1.0)	0.11
Male	1.12 (0.46–2.73)	0.80
Smoking	1.30 (0.51–3.30)	0.58
Alcohol	0.93 (0.37–2.31)	0.88
Lymphedema	0.57 (0.19–1.74)	0.33
Mucous Membranes Involved	0.66 (0.15–2.91)	0.59
CD4 Count	0.99 (0.99–1.0)	0.42
HIV Viral Load Detected	1.78 (0.73–4.31)	0.20
KSHV Detected in PBMC	2.27 (0.50–10.3)	0.29
Anti-KSHV Antibody Titers	1.0 (0.99–1.0)	0.63

## Data Availability

The datasets used for this study are available from the corresponding author upon reasonable request.

## Appendix A

**Table A1:** Baseline characteristics of study participants in the subgroup for T cell analysis.

	No Recurrence (N = 29)	Recurrence (N = 6)	*p* Value
Median age (years)	39 [33–46]	32.5 [32–38]	0.24
Males	55.2%	83.30%	0.20
Smoking	10.3%	16.70%	0.66
Alcohol	24.1%	33.30%	0.64
HIV duration (months)	14 [9–36]	22 [14–48]	0.39
Lymphedema present	11/27 (40.7%)	83.30%	0.059
Median follow-up time (days)	364 [175–448]	178.5 [56–240]	0.076
